# Measurement of Digital Literacy Among Older Adults: Systematic Review

**DOI:** 10.2196/26145

**Published:** 2021-02-03

**Authors:** Sarah Soyeon Oh, Kyoung-A Kim, Minsu Kim, Jaeuk Oh, Sang Hui Chu, JiYeon Choi

**Affiliations:** 1 Mo-Im Kim Nursing Research Institute, College of Nursing, Yonsei University Seoul Republic of Korea; 2 Department of Nursing, Yeoju Institute of Technology Yeoju, Gyeonggi-do Republic of Korea; 3 College of Nursing, Yonsei University Seoul Republic of Korea

**Keywords:** healthy aging, eHealth, telehealth, mobile health, digital literacy, ehealth literacy, aging, elderly, older adults, review, literacy

## Abstract

**Background:**

Numerous instruments are designed to measure digital literacy among the general population. However, few studies have assessed the use and appropriateness of these measurements for older populations.

**Objective:**

This systematic review aims to identify and critically appraise studies assessing digital literacy among older adults and to evaluate how digital literacy instruments used in existing studies address the elements of age-appropriate digital literacy using the European Commission’s Digital Competence (DigComp) Framework.

**Methods:**

Electronic databases were searched for studies using validated instruments to assess digital literacy among older adults. The quality of all included studies was evaluated using the Crowe Critical Appraisal Tool (CCAT). Instruments were assessed according to their ability to incorporate the competence areas of digital literacy as defined by the DigComp Framework: (1) information and data literacy, (2) communication and collaboration, (3) digital content creation, (4) safety, and (5) problem-solving ability, or attitudes toward information and communication technology use.

**Results:**

Searches yielded 1561 studies, of which 27 studies (17 cross-sectional, 2 before and after, 2 randomized controlled trials, 1 longitudinal, and 1 mixed methods) were included in the final analysis. Studies were conducted in the United States (18/27), Germany (3/27), China (1/27), Italy (1/27), Sweden (1/27), Canada (1/27), Iran (1/27), and Bangladesh (1/27). Studies mostly defined older adults as aged ≥50 years (10/27) or ≥60 years (8/27). Overall, the eHealth Literacy Scale (eHEALS) was the most frequently used instrument measuring digital literacy among older adults (16/27, 59%). Scores on the CCAT ranged from 34 (34/40, 85%) to 40 (40/40, 100%). Most instruments measured 1 or 2 of the DigComp Framework’s elements, but the Mobile Device Proficiency Questionnaire (MDPQ) measured all 5 elements, including “digital content creation” and “safety.”

**Conclusions:**

The current digital literacy assessment instruments targeting older adults have both strengths and weaknesses, relative to their study design, administration method, and ease of use. Certain instrument modalities like the MDPQ are more generalizable and inclusive and thus, favorable for measuring the digital literacy of older adults. More studies focusing on the suitability of such instruments for older populations are warranted, especially for areas like “digital content creation” and “safety” that currently lack assessment. Evidence-based discussions regarding the implications of digitalization for the treatment of older adults and how health care professionals may benefit from this phenomenon are encouraged.

## Introduction

### Background

Adopting digital technology is becoming imperative for all areas of service and business including health care. In the era of global aging, digital technology is viewed as a new opportunity to overcome various challenges associated with aging, such as reduced physical and cognitive function, multiple chronic conditions, and altered social networking [1]. Consistent with this trend, the proportion of older populations using digital technology has increased exponentially [2], although this proportion is still smaller than that of younger generations. According to the latest Digital Economy Outlook Report from the Organization for Economic Cooperation and Development (OECD), 62.8% of 55–74-year-olds are now connected to the internet, as are 96.5% of 16–24-year-olds [3].

Improving the inclusion and engagement of older adults in digital technology is becoming increasingly important for the promotion of their health and function [4]. While numerous studies have measured the digital literacy of younger generations [5,6], few have examined the inclusion of older adults in the research and design of digital technologies. Moreover, existing measures of digital literacy for older adults are generally focused on acceptance models and barriers to adoption [7-9], which fail to consider heterogeneity in user ability. As emphasized by Mannheim et al [10], designs that focus heavily on barriers may be marginalizing older adults by assuming that they are less capable of utilizing digital technologies than their younger counterparts.

For health care professionals, the rapid digitalization of social and health care services has various implications for providing older adults with improved access, knowledge, and behavior [11]. Telehealth platforms are a solution for frailer, older adults to receive medical support remotely [12], while GPS can be used to mine personalized data to locate older patients and track or predict their needs [13]. Internet use is associated with reduced likelihood of depression among the retired, and social networking sites represent an opportunity for older adults to reduce feelings of loneliness through online interactions with family and friends [14]. The increasing number of Alzheimer’s disease forums on the microblogging system, Twitter, for example, shows how social networking systems serve as a platform for older individuals to share the latest health-related information with others [13].

Quantifying the digital literacy of older adults is the first step to assist older adults to take advantage of this trend of digitalization in health care. However, when measuring digital literacy among older adults, measures must consider how basic competencies among one age cohort can be harder to achieve for another cohort with fewer information-and-communication-technology experiences and opportunities [15]. In the case of older adults, other age-related factors including life transitions, personal health, attitudes, and economic incentives must also be considered during instrument research and design [16].

### Prior Work

To our knowledge, few systematic reviews to date have evaluated instruments of digital literacy for older adults in general, although 1 systematic review of digitally underserved populations attributed poor eHealth literacy to age, as well as language, educational attainment, residential area, and race [17]. Furthermore, the compatibility between these instruments and older adults has not been measured according to a validated framework.

### Goal of This Study

Therefore, this systematic review aimed to (1) identify and critically appraise studies that involved the assessment of digital literacy among older adults and (2) evaluate how digital literacy instruments used in existing studies address the elements of age-appropriate digital literacy using the European Commission’s Digital Competence (DigComp) Framework [18]. According to DigComp, digital literacy is defined in 5 areas: (1) information and data literacy, (2) communication and collaboration, (3) digital content creation, (4) safety, and (5) problem solving [18]. For this review, we chose the DigComp over other frameworks, such as the International Computer and Information Literacy Study [19] and OECD’s Program for the International Assessment of Adult Competencies [20] because the DigComp Framework is the most generalizable across different regions [21] and age groups [15].

## Methods

### Search Strategy and Data Sources

This systematic review was conducted by searching multiple electronic databases according to Preferred Reporting Items for Systematic Reviews and Meta-Analyses (PRISMA) guidelines [22]. Electronic databases and search engines employed in the initial screening period included PubMed, CINAHL, Embase, and MeSH. The combination of search keywords for each database was summarized in a table (See Multimedia Appendix 1). Keywords were matched to database-specific indexing terms, and searches were not limited to a specific region or study design. However, we limited the year of study to those that were conducted after 2009 for a more recent conceptualization of digital literacy.

The reference lists of identified studies were manually reviewed by a team of academics to prevent relevant studies from being excluded in our search for relevant articles. EndNote X9 was used for database management.

### Eligibility Criteria

We included studies that (1) were published in English, (2) targeted older adults, and (3) measured the use of a validated instrument to assess digital literacy. However, publications were excluded if older adults were not the study’s main target population. To elaborate, publications targeting the general population, for example, were excluded from our list of eligible articles as older adults were not the main target population examined.

Exceptions to this rule were studies that compared older populations to younger populations with the aim of addressing the age-related digital divide, like the study by Schneider and colleagues [23] comparing the digital literacy of “baby boomers” (50-65 years old) to that of millennials (18-35 years old).

### Study Selection

Using these eligibility criteria, 3 independent investigators (SO, MK, and JO) examined all studies reporting the use of a digital literacy instrument in the databases and search engines. All studies were screened according to their title and excluded if the main target population did not consist of older adults.

Subsequently, abstracts were screened so that non-English studies and studies not assessing digital literacy through a validated instrument could be excluded from our investigation. During this process, any studies that were incapable of providing information on the required general characteristics were excluded.

Last, full-text reviews were performed to ensure that all articles measured the digital literacy of older adults through validated instruments. In this process, investigator-developed questionnaires were included only if authors mentioned that they had been evaluated by experts for face validity. The instruments mentioned in each article were checked to ensure that they were accessible for our quality assessment. All processes were supervised by 2 independent reviewers (SC and JC), and any disagreement was resolved through discussions.

### Data Collection

Data on the general characteristics of the included studies included a summary of the year of publication, study design, region where the study was conducted, age of older adults studied, and main literacy instrument used. Regarding the region where the study was conducted, 2 studies were international collaborations, including 1 study between Italy and Sweden [24] and another study between the United States, United Kingdom, and New Zealand [25]. For these 2 studies, the first author’s region of study was used in our general characteristics summary.

### Quality Assessment

Three independent reviewers (SO, JC, and KK) assessed the quality of each included study using the Crowe Critical Appraisal Tool (CCAT) [26]. The CCAT is a validated quality assessment tool developed to rate research papers in systematic reviews based on a number of criteria relative to research design, variables and analysis, sampling methods, and data collection (Multimedia Appendix 2) [26]. Many systematic reviews targeting older adults have used this tool [27,28] for quality appraisal.

Instruments were also assessed to the DigComp’s definition of the 5 areas of digital literacy: (1) information and data literacy (browsing, searching, filtering data), (2) communication and collaboration (interacting, sharing, engaging in citizenship, collaborating), (3) digital content creation (developing, integrating, and re-elaborating digital content; copyright; licenses; programming), (4) safety (protecting devices, protecting personal data and privacy, protecting health and well-being), and (5) problem solving (solving technical problems, identifying needs and technological responses, creatively using digital technologies, identifying digital competence gaps) [18].

## Results

**Figure 1:**
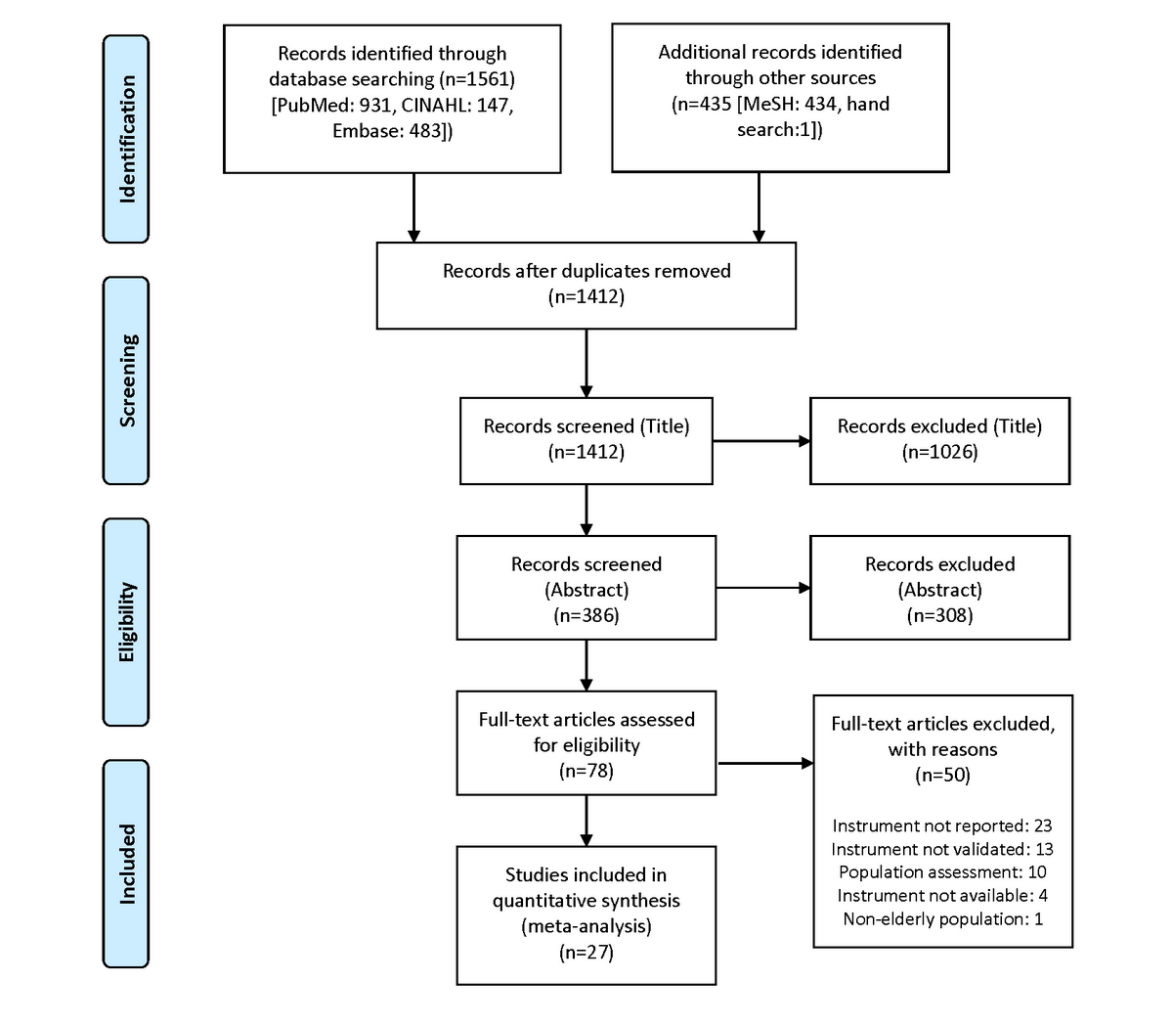
PRISMA flowchart of the literature search and study selection process.

The PRISMA flow diagram in Figure 1 summarizes the search results and selection process of all studies included in our synthesis. Overall, the number of records identified in our database was 1561 (PubMed: 931; CINAHL: 147; Embase: 483). The number of additional records identified through other sources was 435 (MeSH: 434, hand search: 1). Of these records, 1412 remained after duplicates were electronically removed. An additional 1026 articles were removed after title screening, and 308 articles were removed after abstract screening.

### Study Characteristics

Of the 78 articles assessed for eligibility, 50 were excluded for the following reasons: (1) no report of an instrument for digital literacy despite the title or abstract of the paper alluding to measures of digital literacy (n=23); (2) the instrument presented was not validated (n=13); (3) studies were mainly on population assessments and measured digital literacy only as part of a wider assessment of multiple factors (n=10); (3) instruments were not available in English or in a publicly accessible format (n=4); and (4) the study did not specifically target older adults (n=1). Ultimately, 27 articles were included in our review.

Table 1 provides a general summary of the included studies. While publication years ranged from 2009 to 2020, most articles reviewed were conducted between 2015 and 2020. The majority (17/27, 63%) of included studies were cross-sectional, but 2 studies were pre- and post-test studies, 2 were randomized controlled trials (RCTs), 1 was longitudinal, and 1 was a mixed-method study with both surveys and focus group interviews. Most studies were conducted in the United States (18/27), but some studies were also conducted in Europe (Germany, 3/27; Italy, 1/27; Sweden, 1/27). Studies mostly defined older adults as aged ≥50 years (10/27) or ≥60 years (8/27).

Table 2 presents the detailed characteristics of all 27 included studies. Overall, the eHealth Literacy Scale (eHEALS) [29] was the most frequently used instrument to measure digital literacy among older adults (16/27, 59%). The Unified Theory of Acceptance and Usage of Technology (UTAUT) was also used by 2 studies from Germany [9,30] and 1 study from Bangladesh [31]. Loyd-Gressard’s Computer Attitude Scale (CAS) was used in 2 studies that focused heavily on computer anxiety and confidence [32,33]. There was not wide variation in the quality of studies assessed via the CCAT, with scores ranging from 34 (34/40, 85%) to 40 (40/40, 100%) of a total of 40 points.

**Table 1:** Summary of included studies (n=27).

Categories		Results, n (%)
**Year of study publication**		
	2009-2010	2 (7)
	2011-2012	3 (11)
	2013-2014	2 (7)
	2015-2016	6 (22)
	2017-2018	9 (33)
	2019-2020	5 (19)
**Study design**		
	Cross-sectional	17 (63)
	Before and after study	2 (7)
	Randomized controlled trial	2 (7)
	Longitudinal	1 (4)
	Mixed methods^a^	1 (4)
**Region where study was conducted**		
	United States	18 (67)
	Germany	3 (11)
	China	1 (4)
	Italy	1 (4)
	Sweden	1 (4)
	Canada	1 (4)
	Iran	1 (4)
	Bangladesh	1 (4)
**Definition of older adults (years)**		
	≥50	10 (37)
	≥55	4 (15)
	≥60	8 (30)
	≥65	5 (19)
**Main health literacy instrument used**		
	eHealth Literacy Scale (eHEALS)	16 (59)
	Unified Theory of Acceptance and Use of Technology (UTAUT)	3 (11)
	Computer Anxiety Scale (CAS)	2 (7)
	Technology Acceptance Model (TAM)	2 (7)
	Swedish Zimbardo Time Perspective Inventory (S-ZTPI)	1 (4)
	Mobile Device Proficiency Questionnaire (MDPQ)	1 (4)
	Everyday Technology Use Questionnaire (ETUQ)	1 (4)
	Attitudes towards Psychological Online Interventions (APOI)	1 (4)

^a^Survey and focus group interviews.

**Table 2:** Characteristics of included studies.

Author	Year	Country	Sample size, n	Design	Study aim	Measure	CCAT^a,b^ score, points (% of total)
Roque et al [34]	2016	United States	109	Cross-sectional	To validate a new tool for measuring mobile device proficiency across the life span by assessing both basic and advanced proficiencies related to smartphone and tablet use	MDPQ^c^, CPQ^d^-12	37 (93)
Zambianchi et al [24]	2019	Italy and Sweden	638	Cross-sectional	To examine the determinants of attitudes towards and use of ICTs^e^ in older adults	S-ZTPI^f^, ATTQ^g^	38 (95)
Schneider et al [23]	2018	Germany	577	RCT^h^	To examine whether there are any differences in use of an online psychological intervention between generational groups based on Deprexis user data, responses on a questionnaire, and data in the EVIDENT study	APOI^i^	40 (100)
Nagle et al [9]	2012	Germany	52	Cross-sectional	To get a better understanding of the factors affecting older adults' intention towards and usage of computers	UTAUT^j^	34 (85)
Yoon et al [33]	2015	United States	209	Cross-sectional	To examine predictors of computer use and computer anxiety in older Korean Americans	CAS^k^	38 (95)
Cherid et al [35]	2020	Canada	401	Cross-sectional	To identify the current level of technology adoption, health, and eHealth literacy among older adults with a recent fracture, to determine if the use of electronic interventions would be feasible and acceptable in this population	eHEALS^l^	39 (98)
Xie and Bo [36]	2011	United States	146	Cross-sectional	To examine the effects of a theory-driven eHealth literacy intervention for older adults	eHEALS	39 (98)
Tennant et al [37]	2015	United States	393	Cross-sectional	To explore the extent to which sociodemographic, social determinants, and electronic device use influence eHealth literacy and use of Web 2.0 for health information among baby boomers and older adults	eHEALS	36 (90)
Hoogland et al [38]	2020	United States	198	Cross-sectional	To examine age differences in eHealth literacy and use of technology devices/HIT^m^ in patients with cancer and characterize receptivity towards using home-based HIT to communicate with the oncology care team	eHEALS	36 (90)
Price-Haywood et al [39]	2017	United States	247	Cross-sectional	To examine relationships between portal usages, interest in health-tracking tools, and eHealth literacy and to solicit practical solutions to encourage technology adoption.	eHEALS	37 (93)
Paige et al [40]	2018	United States	830	Cross-sectional	To examine the structure of eHEALS scores and the degree of measurement invariance among US adults representing the following generations: millennials (18-35 years old), Generation X (36-51 years old), baby boomers (52-70 years old), and the silent generation (71-84 years old)	eHEALS	38 (95)
Aponte et al [41]	2017	United States	20	Cross-sectional	To explore the experiences of older Hispanics with type 2 diabetes in using the internet for diabetes management	eHEALS	37 (93)
Xie and Bo [42]	2011	United States	124	Cross-sectional	To generate scientific knowledge about the potential impact of learning methods and information presentation channels on older adults' eHealth literacy	eHEALS	39 (98)
Sudbury-Riley and Lynn [25]	2017	United States, United Kingdom, New Zealand	996	Cross-sectional	To examine the factorial validity and measurement invariance of the eHEALS among baby boomers in the United States, the United Kingdom, and New Zealand who had used the internet to search for health information in the last 6 months	eHEALS	35 (88)
Noblin et al [43]	2017	United States	181	Cross-sectional	To determine the willingness of older adults to use health information from a variety of sources	eHEALS	38 (95)
Cajita et al [44]	2018	United States	129	Cross-sectional	To examine factors that influence intention to use mobile technology in health care (mHealth) among older adults with heart failure	TAM^n^	37 (93)
Lin et al [45]	2019	Iran	468	Longitudinal	To examine the temporal associations between eHealth literacy, insomnia, psychological distress, medication adherence, quality of life, and cardiac events among older patients with heart failure	eHEALS	39 (98)
Chu et al [32]	2009	United States	137	RCT	To measure the psychosocial influences of computer anxiety, computer confidence, and computer self-efficacy in older adults at 6 meal congregate sites	CAS	40 (100)
Rosenberg et al [46]	2009	Sweden	157	Cross-sectional	To measure the perceived difficulty in everyday technology use such as remote controls, cell phones, and microwave ovens by older adults with or without cognitive deficits	ETUQ^o^	37 (93)
Stellefson et al [47]	2017	United States	283	Cross-sectional	To examine the reliability and internal structure of eHEALS data collected from older adults aged ≥50 years responding to items over the telephone	eHEALS	36 (90)
Chung et al [48]	2015	United States	866	Cross-sectional	To test the psychometric aspects of the eHEALS for older adults using secondary data analysis	eHEALS	36 (90)
Li et al [49]	2020	China	1201	Cross-sectional	To examine the associations among health-promoting lifestyles, eHealth literacy, and cognitive health in older adults	eHEALS	37 (93)
Choi et al [29]	2013	United States	980	Mixed methods	To examine internet use patterns, reasons for discontinued use, eHealth literacy, and attitudes toward computer or internet use among low-income homebound individuals aged ≥60 years in comparison to their younger counterparts (homebound adults <60 years old)	eHEALS, ATC/IQ^p^	37 (93)
Moore et al [8]	2015	United States	30	Cross-sectional	To offer design considerations in developing internet-based hearing health care for older adults by analyzing and discussing the relationship between chronological age, computer skills, and the acceptance of internet-based hearing health care	TAM	35 (88)
Hoque et al [31]	2017	Bangladesh	300	Cross-sectional	To develop a theoretical model based on the UTAUT and then empirically test it to determine the key factors influencing elderly users’ intention to adopt and use mHealth services	UTAUT	36 (90)
Niehaves et al [30]	2014	Germany	150	Cross-sectional	To study the intentions of the elderly with regard to internet use and identify important influencing factors	UTAUT	35 (88)
Aponte et al [41]	2017	United States	100	Cross-sectional	To examine the validity of the Spanish version of the eHEALS with an older Hispanic population from a number of Spanish-language countries living in New York City	eHEALS	37 (93)

^a^CCAT: Crowe Critical Appraisal Tool.

^b^Total CCAT score is 40 points.

^c^MDPQ: Mobile Device Proficiency Questionnaire.

^d^CPQ: Computer Proficiency Questionnaire.

^e^ICTs: information and communication technologies.

^f^S-ZTPI: Swedish Zimbardo Time Perspective Inventory.

^g^ATTQ: Attitudes Toward Technologies Questionnaire.

^h^RCT: randomized controlled trial.

^i^APOI: Attitudes towards Psychological Online Interventions.

^j^UTAUT: Unified Theory of Acceptance and Use of Technology.

^k^CAS: Computer Attitude Scale.

^l^eHEALS: eHealth Literacy Scale.

^m^HIT: health information technology.

^n^TAM: Adapted Technology Acceptance Model.

^o^ETUQ: Everyday Technology Use Questionnaire.

^p^ATC/IQ: Attitudes Toward Computer/Internet Questionnaire.

As seen in Table 3, all instruments were analyzed for quality assessment to assess which DigComp elements of digital literacy were met [18]. Studies mostly satisfied 1 or 2 aspects of the information and data literacy criteria, but the Mobile Device Proficiency Questionnaire (MDPQ) satisfied all 5 elements, including those related to safety and data creation.

**Table 3:** Inclusion of the European Commission’s Digital Competence (DigComp) Framework criteria and quality assessment of the included studies.

Measure	Literacy elements^a^					Mode	Scoring	Reliability, Cronbach α
	1	2	3	4	5			
Attitude Toward Technologies Questionnaire (ATTQ)	O^b^	O	X^c^	X	X	Self-administered	6 5-point Likert questions	0.91 (Italy), 0.92 (Sweden) [50]
Adapted Technology Acceptance Model (TAM)	O	O	X	X	X	Self-administered	6 7-point Likert questions	0.91 (perceived ease of use), 0.97 (perceived usefulness), 0.96 (attitude toward using), 0.70 (actual system use) [51]
Attitudes Toward Computer/Internet Questionnaire (ATC/IQ)	O	X	X	X	X	Interview (semistructured)	10 5-point Likert questions	0.98 (usefulness), 0.94 (ease of use) [52], adapted by Choi and DiNitto [29]
Attitudes Towards Psychological Online Intervention Questionnaire (APOI)	O	X	X	X	X	Self-administered	16 5-point Likert questions	0.77 (total), 0.62 (skepticism and perception of risks), 0.62 (anonymity benefits), 0.64 (technologization threat), 0.72 (confidence in effectiveness) [53]
Computer Attitude Scale (CAS)	X	X	X	O	O	Self-administered	4 10-point Likert questions	0.95 (total), 0.90 (computer anxiety), 0.89 (computer confidence), 0.89 (computer liking), 0.82 (computer usefulness) [54]
eHealth Literacy Scale (eHEALS)	O	O	X	O	O	Self-administered	8 5-point Likert questions	0.88, 0.60-0.84 (range among items) [55]
Computer Proficiency Questionnaire (CPQ)	O	O	O	X	X	Self-administered	33 5-point Likert questions	0.98 (total for CPQ) 0.95 (total for CPQ-12), 0.91 (computer basics), 0.94 (printing), 0.95 (communication), 0.97 (internet), 0.96 (scheduling), 0.86 (multimedia) [56]
Mobile Device Proficiency Questionnaire (MDPQ)	O	O	O	O	O	Self-administered	46 5-point Likert questions	0.75 (MDPQ-46), 0.99 (MDPQ-16) [34]
Unified Theory of Acceptance and Usage of Technology (UTAUT)	O	O	O	X	O	Interview (face-to-face)	15 7-point Likert questions	0.7879-0.9497 [57]

^a^European Commission’s Digital Competence (DigComp) Framework criteria of (1) information and data literacy (browsing, searching, filtering data), (2) communication and collaboration (interacting, sharing, engaging in citizenship, collaborating), (3) digital content creation (developing, integrating, and re-elaborating digital content; copyright; licenses; programming), (4) safety (protecting devices, protecting personal data and privacy, protecting health and well-being), and (5) problem solving (solving technical problems, identifying needs and technological responses, creatively using digital technologies, identifying digital competence gaps) [18].

^b^O: included in the questionnaire.

^c^X: not included in the questionnaire.

## Discussion

### Principal Findings

In this systematic review, we highlighted the importance of digital literacy among older adults and provided a comprehensive overview of the instruments that are being employed to measure their digital literacy. We also illustrated the various strengths and limitations of each instrument, relative to age-appropriateness and suitability for older adults, in accordance with the components of a validated, digital competency framework [18]. Our review is timely because, to the best of our knowledge, few systematic reviews to date have evaluated measurements of digital literacy for older adults specifically.

In the digital era, providing education for patients regarding management of their physical or mental illness or injury, explaining posttreatment home care needs, and managing their diet, nutrition, and exercise are all duties that are beginning to be “digitalized” [58]. Moreover, digital technologies are providing practitioners with more effective and user-centered ways to educate, inform, and treat older patients. For example, in a systematic review of “virtual visits” in home care for older patients, both service users and providers found online visits to be more flexible, easy to arrange, and personal than offline visits [59]. In another study of an internet-based videoconferencing system for frail elderly people in Nordic countries, telehealth was associated with reduced loneliness among 88% of users, while simultaneously reducing the odds of catching a cold during winter months due to leaving the house [60].

Overall, we discovered that while the eHEALS is most frequently used to measure digital literacy among older adults, the MDPQ may be more appropriate for measuring the literacy of older adults. Unlike the eHEALS, the MDPQ attempts to measure older adults’ digital content creation capacity (developing, integrating, and re-elaborating digital content; copyright; licenses; programming), which according to the European Commission, can give valuable information regarding an individual’s ability to add value to new media for self-expression and knowledge creation [18].

Also, the MDPQ contains numerous items related to data protection and privacy such as “passwords can be created to block/unblock a mobile device” or “search history and temporary files can be deleted” despite the fact that security was the least measured element of the DigComp Framework among the instruments in our study. Only the CAS, eHEALS, and MDPQ provide items related to data protection and privacy, which is concerning given that older adults comprise a significant proportion of the target population for internet scams or email attacks [61].

In our review of 27 selected articles, more than half (16/27, 59%) used the eHEALS to measure the digital literacy of older adults. Several reasons can be speculated; this instrument is short (8 items), and the questions are simple to understand (eg, “I know how to use the Internet to answer my health questions”). Scholars claim that it is easy to administer to older adults [48]. It should be noted that because of its simplicity, there has been some debate regarding the validity of the eHEALS [62-64]. As described by Jordan and colleagues [64], the eHEALS has a “lack of an explicit definition of the concept that many health literacy indices were developed to measure limit... ...its ability to make fully informed judgments... ...about a person’s ability to seek, understand, and use health information.”

Studies focusing on similar research aims also employ similar instruments. For example, the CAS was used in 2 studies that focused on computer anxiety and confidence. In the existing body of literature, the CAS has often been used for studies targeting individuals in highly stressful environments such as business graduate students [65], psychiatric inpatients [66], and students studying at a 2-year technical college experiencing “technostress” [67]. As explained by Kelley and Charness [68], older adults “commit more errors in post-training evaluations” than the general population, which may result in greater stress and anxiety. This may demonstrate the suitability of the CAS for older adult populations.

Regarding the overall quality of the included studies evaluated using the CCAT, some variation existed among the studies reviewed. Studies that were cross-sectional or lacked acquisition of written informed consent and used alternate approaches, such as telephone or self-reported, web-based or email surveys, scored poorly in the “design” and “ethical matters” category. Studies also lost marks if there was no flow diagram, there was no mention of design methods in the title of their manuscript, or they had biased sampling methods (convenience sampling, pertaining only to 1 or 2 ethnic groups).

Contrastingly, 2 RCTs in our review received a score of 100% on the CCAT, as they had excellent preliminaries, introductions, study design, sampling methods, data collection methods, ethical matters, results, and discussions. These studies employed performance-based measures like the Attitudes Toward Computer/Internet Questionnaire (ATC-IQ; semistructured interview) and UTAUT model (face-to-face interview), which are more reliable data collection methods than self-administered questionnaires. Performance-based measures like these may be suitable for studies targeting older adults, but it should be noted that clinical environments and personal fitness can greatly influence outcomes, especially if environments contain learners of mixed ability [69], rapid progression [34], and the possibility for embarrassment or discomfort [70]. Positive clinical settings are associated with improved performance, as observed in 1 of the RCTs in our review, where “a combination of patience, perseverance, and peer-to-peer or instructor encouragement, whether with words or a pat on the shoulder” were successful in reducing older adults’ stress and anxiety during digital learning [32].

As aforementioned, for older adults, it is important that the research and design of digital technologies encompass the heterogeneity of their capacity. While we believe that instructions should be “clear and understandable” to study participants [34], we also believe that literacy elements that are generalizable to the rest of the population (relative to communication, safety, problem solving, and competence) should be measured for this population as well. As described by Hänninen et al [16], the digital capacity of older adults lies on a continuum or spectrum and can range from actively independent to limited.

Previous studies recommend that, instead of employing the full MDPQ or technology acceptance model (TAM), the shorter, 16-question version [34], or senior version of the TAM (Senior Technology Acceptance & Adoption Model), may be more appropriate for relatively older and frailer populations [7]. User-centeredness in instrument development and measurement is crucial for this population, as the functional status of older adults varies immensely. Furthermore, scales and scoring methods are encouraged to be as inclusive as possible, so that they encompass the diversity in functionality that exists among study subjects.

### Limitations

Ultimately, many limitations exist in our review. First, it is important to mention that the association between age and digital capacity is controversial among certain scholars who argue that age-based divisions are too simplistic [23] and unclear [71] to explain the digital divide. In the Netherlands, for example, “digital natives” do not appear to exist, and other factors like life stages and socialization are considered to be more relevant proxies of digital literacy than age [71]. Also, in a German study, perceptions of threat due to technologization were perceived as the main predictors of digital capacity, rather than age itself [23]. Older adults with lower perceptions of threat could be digitally fluent, just as younger adults with higher perceptions of threat could be digitally illiterate. Future questionnaires should consider measuring this factor in depth and the possible interaction that it has with age in predicting digital capacity outcomes.

Likewise, digital literacy is a process-oriented skill, and measuring it in isolation may be inaccurate for quantifying an individual’s skillset [72]. In the Lily Model, Norman and Skinner [72] posit that there are 6 core skills of literacy: traditional, media, information, computer, scientific, and health. Not only are these skills heavily interconnected with one another but also only an in-depth analysis of all 6 can fully contextualize an individual’s personal, social, and environmental contexts [72]. For example, computer literacy may be heavily influenced by an individual’s ability to understand and read passages (traditional literacy) as well as their ability to find information on a topic (information literacy) and understand certain scientific terms (science literacy). Because these literacy types are interconnected, only an in-depth analysis of all 6 may accurately measure an individual’s knowledge.

Also, as observed in our review, many of the investigated instruments, including the Attitudes Toward Technologies Questionnaire, TAM, ATC-IQ, APOI, and CAS, measured attitudes or perceptions toward technology rather than digital aptitude itself. While studies on attitude are important, the lack of measures examining older adults’ abilities to use information and communications technology was an unexpected limitation of the reviews studied.

Last, even though previous studies have argued that the DigComp Framework is one of the broadest and most generalizable frameworks for assessing digital literacy measures [15,21], it is undeniable that certain types of survey error are more likely to occur among older populations relative to memory loss, health problems, sensory and cognitive impairments, and personal or motivational factors that influence their ability to participate in an investigation [73]. The author and editors of this framework specifically mention in their proposal that, because they adopted a “general” rather than “individual” approach, their framework should be considered only as a starting point in interpreting digital competence among different age groups [18].

### Conclusions

In conclusion, more studies are required so that the measurement of digital literacy among older adults can become more elaborate and specific. Digital literacy evidently has strong associations with the utility of information and communications technologies that promote physical and mental well-being among older adults. Further assessments and studies of digital literacy among older adults that overcome the limitations of existing research and measurement designs would allow for better allocation of support and resources to address the diverse health care needs of this growing but vulnerable population.

## Appendix

Multimedia Appendix 1 Summary of database (DB) search terms.

Multimedia Appendix 2 Crowe Critical Appraisal Tool (CCAT) form.

## Abbreviations

APOI: Attitudes towards Psychological Online Interventions
ATC-IQ: Attitudes Toward Computer/Internet Questionnaire
CAS: Computer Attitude Scale
CCAT: Crowe Critical Appraisal Tool
DigComp: European Commission’s Digital Competence framework
eHEALS: eHealth Literacy Scale
MDPQ: Mobile Device Proficiency Questionnaire
OECD: Organization for Economic Cooperation and Development
PRISMA: Preferred Reporting Items for Systematic Reviews and Meta-Analyses
RCT: randomized controlled trial
TAM: technology acceptance model
UTAUT: unified theory of acceptance and usage of technology
