# Correction: Measurement of Digital Literacy Among Older Adults: Systematic Review

**DOI:** 10.2196/28211

**Published:** 2021-03-03

**Authors:** Sarah Soyeon Oh, Kyoung-A Kim, Minsu Kim, Jaeuk Oh, Sang Hui Chu, JiYeon Choi

**Affiliations:** 1 Mo-Im Kim Nursing Research Institute, College of Nursing, Yonsei University Seoul Republic of Korea; 2 Department of Nursing, Yeoju Institute of Technology Yeoju, Gyeonggi-do Republic of Korea; 3 College of Nursing, Yonsei University Seoul Republic of Korea

In the originally published paper, the footnotes under Table 3 were incorrect.

The footnotes originally appeared as follows:

^b^O: not included in the questionnaire

^c^X: included in the questionnaire

These have now been corrected to the following:

^b^O: included in the questionnaire

^c^X: not included in the questionnaire

The correction will appear in the online version of the paper on the JMIR Publications website on March 03, 2021, together with the publication of this correction notice. Because this was made after submission to PubMed, PubMed Central, and other full-text repositories, the corrected article has also been resubmitted to those repositories.

