# Correction: Measurement of Digital Literacy Among Older Adults: Systematic Review

**DOI:** 10.2196/30828

**Published:** 2021-06-15

**Authors:** Sarah Soyeon Oh, Kyoung-A Kim, Minsu Kim, Jaeuk Oh, Sang Hui Chu, JiYeon Choi

**Affiliations:** 1 Mo-Im Kim Nursing Research Institute, College of Nursing, Yonsei University Seoul Republic of Korea; 2 Department of Nursing, Yeoju Institute of Technology Yeoju, Gyeonggi-do Republic of Korea; 3 College of Nursing, Yonsei University Seoul Republic of Korea

In “Measurement of Digital Literacy Among Older Adults: Systematic Review” (J Med Internet Res 2021;23(2):e26145), the authors noted one error.

In the originally published paper, the funding statement appeared as follows:

This research was supported by the Brain Korea 21 FOUR Project funded by the National Research Foundation of Korea, Yonsei University College of Nursing. This study received funding from the National Research Foundation of Korea (grant number 2020R1A6A1A0304198911 [SO, KK, and JC]), the Ministry of Education of the Republic of Korea and the National Research Foundation of Korea (NRF-2017S1A3A2067165 [SC]), and Yonsei University College of Nursing Faculty Research Fund (6-2020-0188 [SC and JC]).

In the corrected version of the paper, the funding statement has been revised as follows:

This research was supported by the Brain Korea 21 FOUR Project funded by the National Research Foundation of Korea, Yonsei University College of Nursing. This study received funding from the National Research Foundation of Korea (grant number 2020R1A6A1A0304198911 [SO, KK, and JC]), the Ministry of Education of the Republic of Korea, and Yonsei University College of Nursing Faculty Research Fund (6-2020-0188 [SHC and JC]).

The correction will appear in the online version of the paper on the JMIR Publications website on June 15, 2021, together with the publication of this correction notice. Because this was made after submission to PubMed, PubMed Central, and other full-text repositories, the corrected article has also been resubmitted to those repositories.

