# Supplementary material for: Measurement of Digital Literacy Among Older Adults: Systematic Review
Source: J Med Internet Res. 2021 Feb 3;23(2):e26145. doi: 10.2196/26145 (PMC7889415; doi:10.2196/26145)
Supplement: Multimedia Appendix 1 [file jmir_v23i2e26145_app1.docx]

| Multimedia Appendix 1. Summary of Database (DB) Search Terms. | |
| --- | --- |
| **DB** | **Keywords** |
| PubMed | ("digital literacy" OR "digital disparity" OR "digital divide" OR "technology literacy" OR "technology disparity" OR "technology divide" OR "mhealth literacy" OR "m-health literacy" OR "ehealth literacy" OR "e-health literacy" OR "computer literacy") AND ("measurement" OR "measure" OR "validity" OR "survey" OR "questionnaire" OR "instrument" OR "screener" OR "tool" OR "psychometric") |
| CINAHL | ("digital literacy" OR "digital disparity" OR "digital divide" OR "technology literacy" OR "technology disparity" OR "technology divide" OR "mhealth literacy" OR "m-health literacy" OR "ehealth literacy" OR "e-health literacy" OR "computer literacy") AND ("measurement" OR "measure" OR "validity" OR "survey" OR "questionnaire" OR "instrument" OR "screener" OR "tool" OR "psychometric") |
| Embase | ('digital literacy' OR 'digital disparity' OR 'digital divide' OR 'technology literacy' OR 'technology disparity' OR 'technology divide' OR 'mhealth literacy' OR 'm-health literacy' OR 'ehealth literacy' OR 'e-health literacy' OR 'computer literacy') AND ('measurement' OR 'measure' OR 'validity' OR 'survey' OR 'questionnaire' OR 'instrument' OR 'screener' OR 'tool' OR 'psychometric') AND ([adult]/lim OR [aged]/lim OR [very elderly]/lim) AND [humans]/lim AND [English]/lim AND [2000-2020]/py |
| MeSH | "Digital Divide"[Mesh] OR "Computer Literacy"[Mesh] |
