# Supplementary figures and images for: Measurement of Digital Literacy Among Older Adults: Systematic Review
Source: J Med Internet Res. 2021 Feb 3;23(2):e26145. doi: 10.2196/26145 (PMC7889415; doi:10.2196/26145)

**Supplementary File 1. CCAT.**

**
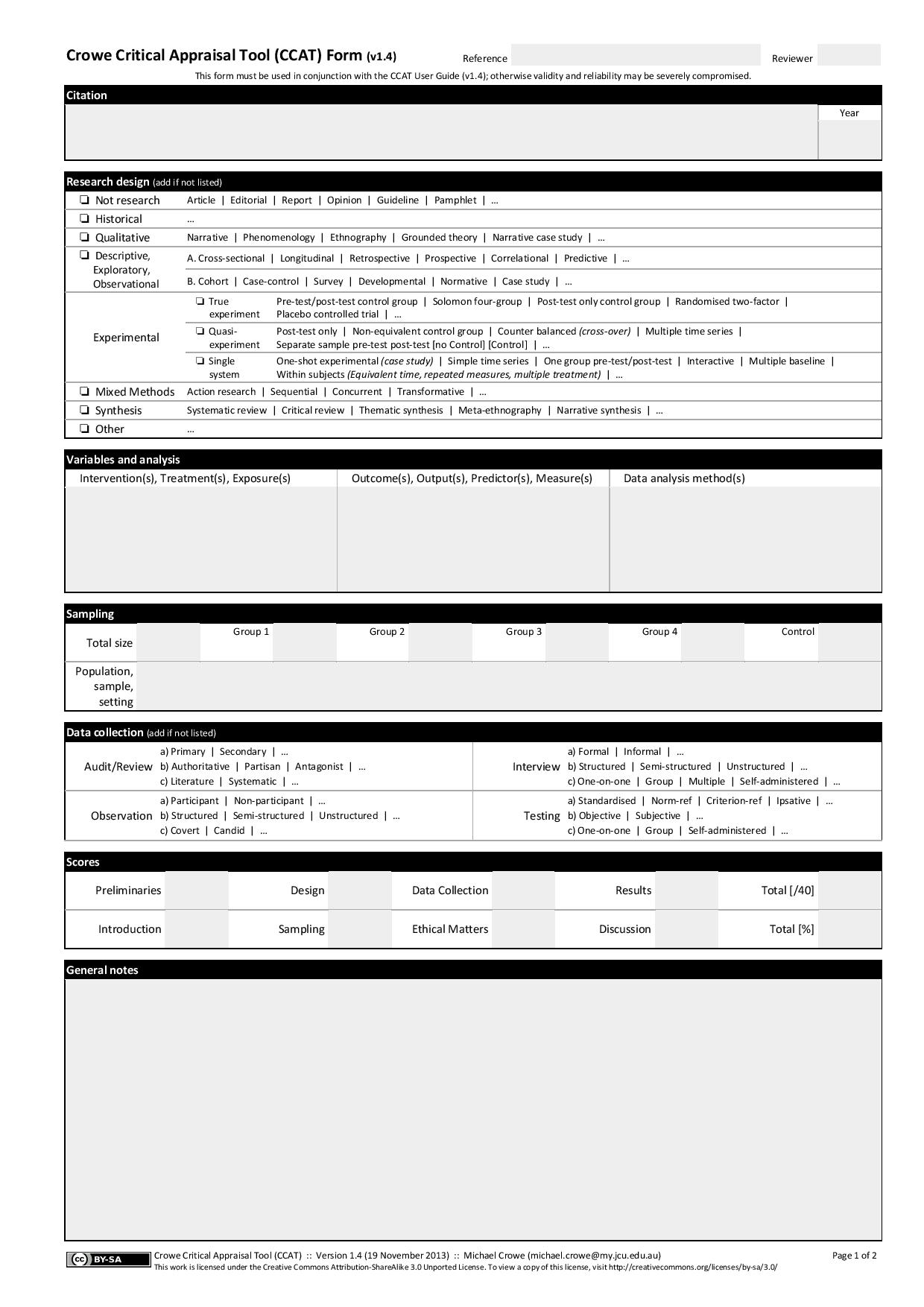
**

**
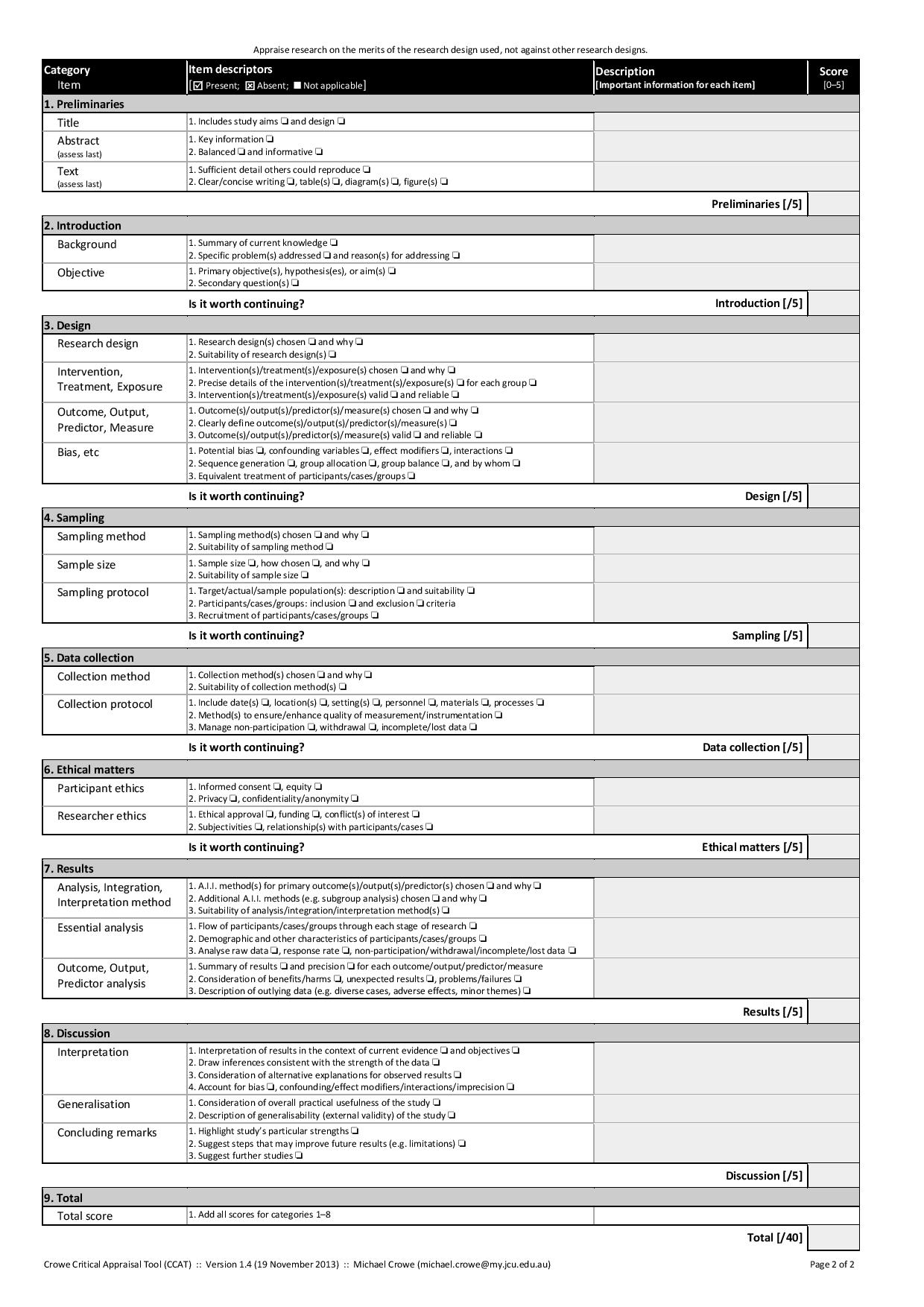
**

Supplement: Multimedia Appendix 2 [file jmir_v23i2e26145_app2.docx]
